# Does somatosensory discrimination therapy alter sensorimotor upper limb function differently compared to motor therapy in children and adolescents with unilateral cerebral palsy: study protocol for a randomized controlled trial

**DOI:** 10.1186/s13063-024-07967-4

**Published:** 2024-02-26

**Authors:** Lize Kleeren, Lisa Mailleux, Belinda McLean, Catherine Elliott, Griet Dequeker, Anja Van Campenhout, Jean-Jacques Orban de Xivry, Geert Verheyden, Els Ortibus, Katrijn Klingels, Hilde Feys

**Affiliations:** 1 KU Leuven, Department of Rehabilitation Sciences, Research Group for Neurorehabilitation, Leuven, B-3001 Belgium; 2 KU Leuven, Child and Youth Institute, Leuven, B-3000 Belgium; 3 Hasselt University, Rehabilitation Research Centre, Faculty of Rehabilitation Sciences, Diepenbeek, B-3590 Belgium; 4 Curtin School of Allied Health, Faculty of Health Sciences, Curtin University, Perth, Australia; 5 University Hospitals Leuven, Cerebral Palsy Reference Centre, Leuven, B-3000 Belgium; 6 KU Leuven, Department of Development and Regeneration, Leuven, B-3000 Belgium; 7 KU Leuven, Leuven Brain Institute, Leuven, B-3000 Belgium; 8 KU Leuven, Department of Movement Sciences, Research Group of Motor Control and Neuroplasticity, Leuven, B-3000 Belgium; 9 Kids Rehab WA, Telethon Kids Institute, Perth, Australia

**Keywords:** Unilateral cerebral palsy, Upper extremity, Somatosensation, Sensorimotor function, Neuroimaging, Bimanual performance, Randomized controlled trial, Physiotherapy, Occupational therapy

## Abstract

**Background:**

Besides motor impairments, up to 90% of the children and adolescents with unilateral cerebral palsy (uCP) present with somatosensory impairments in the upper limb. As somatosensory information is of utmost importance for coordinated movements and motor learning, somatosensory impairments can further compromise the effective use of the impaired upper limb in daily life activities. Yet, intervention approaches specifically designated to target these somatosensory impairments are insufficiently investigated in children and adolescents with uCP. Therefore, the aim of this randomized controlled trial (RCT) is to compare the effectiveness of somatosensory discrimination therapy and dose-matched motor therapy to improve sensorimotor upper limb function in children and adolescents with uCP, who experience somatosensory impairments in the upper limb. We will further explore potential behavioral and neurological predictors of therapy response.

**Methods:**

A parallel group, evaluator-blinded, phase-II, single-center RCT will be conducted for which 50 children and adolescents with uCP, aged 7 to 15 years, will be recruited. Participants will be randomized to receive 3 weekly sessions of 45 minutes of either somatosensory discrimination therapy or upper limb motor therapy for a period of 8 weeks. Stratification will be performed based on age, manual ability, and severity of tactile impairment at baseline. Sensorimotor upper limb function will be evaluated at baseline, immediately after the intervention and after 6 months follow-up. The primary outcome measure will be bimanual performance as measured with the Assisting Hand Assessment. Secondary outcomes include a comprehensive test battery to objectify somatosensory function and measures of bimanual coordination, unimanual motor function, and goal attainment. Brain imaging will be performed at baseline to investigate structural brain lesion characteristics and structural connectivity of the white matter tracts.

**Discussion:**

This protocol describes the design of an RCT comparing the effectiveness of somatosensory discrimination therapy and dose-matched motor therapy to improve sensorimotor upper limb function in children and adolescents with uCP. The results of this study may aid in the selection of the most effective upper limb therapy, specifically for children and adolescents with tactile impairments.

**Trial registration:**

ClinicalTrials.gov (NCT06006065). Registered on August 8, 2023.

**Supplementary Information:**

The online version contains supplementary material available at 10.1186/s13063-024-07967-4.

## Administrative information

Note: the numbers in curly brackets in this protocol refer to SPIRIT checklist item numbers. The order of the items has been modified to group similar items (see http://www.equator-network.org/reporting-guidelines/spirit-2013-statement-defining-standard-protocol-items-for-clinical-trials/).

**Table:** 

Title {1}	Does somatosensory discrimination therapy alter sensorimotor upper limb function differently compared to motor therapy in children and adolescents with unilateral cerebral palsy: study protocol for a randomized controlled trial
Trial registration {2a and 2b}.	Primary registry and trial identifying number: ClinicalTrials.gov, NCT06006065 Date of registration in primary register: August 8, 2023 Secondary identifying numbers: G0C4919N, S67467 Source(s) of monetary or material support: The Flemish Research Foundation provides financial support for this study (FWO project, G0C4919N; FWO-fellowship Lize Kleeren, 11PP224N). Primary sponsor: KU Leuven Contact for public queries: Lize Kleeren; Herestraat 49, box 1510, B-3000 Leuven, Belgium; lize.kleeren@kuleuven.be ; + 32 16 37 79 10 Contact for scientific queries: PI: Hilde Feys, Scientific contact delegate: Lize Kleeren; Herestraat 49, box 1510, B-3000 Leuven, Belgium; lize.kleeren@kuleuven.be ; + 32 16 37 79 10 Public title: The effectiveness of an intensive therapy program for the upper limb in children and adolescents with unilateral cerebral palsy Scientific title: The effectiveness of an intensive upper limb therapy program focused on somatosensation in children and adolescents with unilateral cerebral palsy Countries of recruitment: Belgium Health condition(s) or problem(s) studied: Unilateral cerebral palsy Intervention(s): Intervention: Upper limb somatosensory discrimination therapy (18 hours over 8 weeks); Active comparator: Upper limb motor therapy (18 hours over 8 weeks) Key inclusion and exclusion criteria: Ages eligible for study: 7 to 15 years; Sexes eligible for study: both; Accepts healthy volunteers: no; Inclusion criteria: (1) Diagnosis of predominantly spastic unilateral cerebral palsy; (2) Aged 7 to 15 years old at the time of baseline assessment; (3) Sufficient cooperation to comprehend and complete the test and therapy procedures; (4) Minimal ability to actively grasp and hold an object with the more impaired hand (Modified House Functional Classification System ≥ 4); and (5) Having a confirmed impairment in tactile registration and/or tactile perception, as objectified using a clinical assessment battery described under the section ‘eligibility screening’; Exclusion criteria: (1) Botulinum toxin-A injections six months prior to baseline testing; (2) A history of upper limb surgery one year prior to baseline testing; and (3) Inability to communicate in Dutch Study type: Type of study: Interventional; Allocation: randomized; Interventional model: parallel assignment; Masking: single blind (assessor); Primary purpose: clinical intervention; Phase: II Date of first enrollment: August 2023 (actual) Target sample size: 50 (anticipated) Recruitment status: Started on August 8, 2023 Primary outcome(s): Outcome name: Bimanual performance; Method of measurement: Assisting Hand Assessment (AHA); Timepoint: 6 months after the therapy has ended (follow-up) Key secondary outcomes: • Outcome name: Bimanual performance; Method of measurement: Assisting Hand Assessment (AHA); Timepoint: immediately after therapy has ended (post-intervention) • Outcome name: Bimanual performance; Method of measurement: Children’s Hand-use Experience Questionnaire (CHEQ); Timepoint: immediately after therapy has ended (post-intervention) and 6 months after the therapy has ended (follow-up) • Outcome name: Bimanual coordination; Method of measurement: Box opening task; Timepoint: immediately after therapy has ended (post-intervention) and 6 months after the therapy has ended (follow-up) • Outcome name: Bimanual coordination; Method of measurement: Kinarm Exoskeleton (‘Ball-on-bar task’); Timepoint: immediately after therapy has ended (post-intervention) and 6 months after the therapy has ended (follow-up) • Outcome name: Tactile registration; Method of measurement: Semmes–Weinstein Monofilaments; Timepoint: immediately after therapy has ended (post-intervention) and 6 months after the therapy has ended (follow-up) • Outcome name: Tactile perception; Method of measurement: Stereognosis, Aesthesiometer, Tactile Discrimination Test; Timepoint: immediately after therapy has ended (post-intervention) and 6 months after the therapy has ended (follow-up) • Outcome name: Proprioception; Method of measurement: Kinarm Exoskeleton (‘Contralateral position matching task’, ‘Perceptual boundary task’, ‘Indicating location task’), ETH MIKE (‘Passive position sense task’), Clinical assessment of movement sense; Timepoint: immediately after therapy has ended (post-intervention) and 6 months after the therapy has ended (follow-up) • Outcome name: Unimanual motor function; Method of measurement: Tyneside Pegboard Test; Timepoint: immediately after therapy has ended (post-intervention) and 6 months after the therapy has ended (follow-up) • Outcome name: Self-perceived occupational performance and goal attainment; Method of measurement: Canadian Occupational Performance Measure (COPM), Goal Attainment Scaling (GAS); Timepoint: immediately after therapy has ended (post-intervention) and 6 months after the therapy has ended (follow-up)
Protocol version {3}	19th of January 2024 – version 5.2
Funding {4}	The Flemish Research Foundation provides financial support for this study (FWO project, G0C4919N; FWO-fellowship Lize Kleeren, 11PP224N).
Author details {5a}	Lize Kleeren^1,2^, Lisa Mailleux^1,2^, Belinda McLean^3^, Catherine Elliott^3^, Griet Dequeker^4^, Anja Van Campenhout^2,4–6^, Jean-Jacques Orban de Xivry^6,7^, Geert Verheyden^1^, Els Ortibus^2,4–6^, Katrijn Klingels^1,8^, Hilde Feys^1,2^ *1 KU Leuven, Department of Rehabilitation Sciences, Research group for Neurorehabilitation, B-3001 Leuven, Belgium* *2 KU Leuven, Child and Youth Institute, B-3000 Leuven, Belgium* *3 Curtin School of Allied Health, Faculty of Health Sciences, Curtin University, Perth, Australia; Kids Rehab WA, Telethon Kids Institute, Perth, Australia* *4 University Hospitals Leuven, Cerebral Palsy Reference Centre, B-3000 Leuven, Belgium* *5 KU Leuven, Department of Development and Regeneration, B-3000 Leuven, Belgium* *6 KU Leuven, Leuven Brain Institute, B-3000 Leuven, Belgium* *7 KU Leuven, Department of Movement Sciences, Research group of Motor Control and Neuroplasticity, B-3000 Leuven, Belgium* *8 Hasselt University, Rehabilitation Research Centre, Faculty of Rehabilitation Sciences, B-3590 Diepenbeek, Belgium* Study conception and design: LK, LM, GD, GV, EO, AVC, KK, HF; Development of proprioceptive assessments: JODX; Conceptualization of the Sense for Kids therapy: BM, CE; Conceptualization of the upper limb motor therapy: LK, LM, GD, HF, KK; Writing of original draft: LK; Reviewing and editing manuscript, Refinement of the study protocol: all authors; Supervision: LM, KK, HF; Funding acquisition: LM, GV, EO, AVC, KK, HF.
Name and contact information for the trial sponsor {5b}	Hilde Feys (KU Leuven); Naamsestraat 22, box 5400, B-3000, Leuven, Belgium; hilde.feys@kuleuven.be ; + 32 16 32 90 79
Role of sponsor {5c}	The funding body, the Flemish Research Organization (FWO), was not involved in the conception or writing of this study protocol. The funding body will not be involved in the collection, management, analysis or interpretation of data. The trial sponsor, UZ/KU Leuven, represented by the principal investigator, was involved in all of the aforementioned processes and has the ultimate authority over the project.

## Introduction

### Background and rationale {6a}

When performing activities of daily living, such as filling a drinking cup or jumping rope, we continuously rely on the process of sensorimotor integration for successful task performance [1]. Sensorimotor integration has been described as the ability to integrate sensory information from various modalities, while simultaneously transforming these inputs into motor actions [2]. More specifically, our somatosensory system provides relevant information on the characteristics of objects, such as their form, weight, and size. Also information regarding the position and movements of our body parts in space is provided, which is critical for a smooth interaction with these objects [1, 2]. As such, somatosensory impairments can significantly disturb the process of sensorimotor integration, leading to difficulties when performing goal-directed activities and exploring the environment [1, 3–5].

This is often the case for children and adolescents with neurological disorders, such as cerebral palsy (CP). CP is the most common physical disability in childhood, arising from an injury or malformation of the developing brain [6, 7]. Unilateral cerebral palsy (uCP) is a prevalent subtype, accounting for up to 44% of the cases [8]. Children and adolescents with uCP present with motor and somatosensory impairments predominantly at one side of the body, which are often more pronounced in the upper compared to the lower limb [9]. Although CP has been primarily defined as a motor disorder, previous research has shown that up to 90% of the children and adolescents with uCP also present with impairments in one or more modalities of somatosensation [10–12].

Somatosensation comprises all aspects of touch and proprioception that contribute to a person’s awareness of his or her body parts in space and the direct interface of these body parts with objects and the environment in the absence of vision [13]. Somatosensory impairments can occur across different modalities of somatosensation, including tactile registration, tactile perception, and proprioception. Tactile registration comprises the awareness of an external stimulus, while tactile perception also includes its interpretation [14]. Proprioception, on the other hand, refers to the ability to perceive limb positions and movements [15]. For example when filling a drinking cup, tactile registration and perception are needed to provide information on the cup’s texture and weight which is needed to adequately tune grip force [16], while proprioception ensures that the position of both hands relative to each other is properly maintained as the cup is filled and its weight increases [17].

As such, accurate somatosensory information is crucial for motor control and motor learning [1, 18, 19]. In children and adolescents with uCP, the presence of somatosensory impairments has shown to be related to worse motor performance, diminished fine motor skills, and reduced use of the impaired upper limb during bimanual activities [10, 20–25]. As a result, developmental experiences will be restricted, limiting somatosensory input and motor development even more [6]. This vicious circle emphasizes the importance of addressing these somatosensory impairments during rehabilitation [26, 27], to improve the use of the more impaired upper limb during bimanual daily life activities potentially further and as such stimulate developmental experiences, functional independence, and quality of life.

Up until now, improvements in somatosensory function are mostly considered as a potential by-product of motor training [28]. Some studies have indeed shown that after constraint-induced movement therapy [29] and bimanual motor therapy [30–32], significant improvements in somatosensory function can be detected, although the results are varying depending on the type of participants, specific intervention performed, and the outcome measures used. Furthermore, based on the training principle of specificity, it can be expected that interventions designated to specifically address these somatosensory impairments might have a superior effect. However, the effectiveness of such intervention approaches has scarcely been investigated in children and adolescents with uCP [28, 33, 34], despite growing evidence in adult stroke patients.

Given the lack of strong evidence regarding somatosensory therapy approaches specifically for children with uCP, Auld and colleagues identified therapy approaches used in adult stroke patients that may also be beneficial for children and adolescents [35]. Transfer enhanced somatosensory discrimination therapy, also known as Sense© therapy, was one of the recommended approaches [36]. Sense© therapy is a highly structured therapy approach based on the principles of perceptual learning and learning-dependent neuroplasticity. It consists of repeated and graded practice discriminating differences in a variety of stimuli across somatosensory modalities, and the practice of daily life activities with targeted attention for somatosensory aspects necessary for successful task completion [37]. The effectiveness of this therapy to improve somatosensory impairments and functional upper limb use in adult stroke patients has been investigated earlier [35, 36, 38–40]. In 2017, McLean and colleagues modified the original concept of this therapy to accommodate the specific needs of children and adolescents, resulting in the Sense for Kids therapy [41]. Based on a feasibility study in children and adolescents with uCP [41], this therapy might improve somatosensory function, goal attainment, and motor performance. Furthermore, Sense for Kids therapy has shown to be feasible and engaging [42]. The full potential and the long-term effects of this therapy in comparison to upper limb motor therapy, however, still need to be investigated in a randomized controlled trial (RCT).

Despite having the same diagnosis, large inter-individual variability with regard to sensorimotor upper limb function and therapy response after motor therapy is seen in children and adolescents with uCP [43–48]. This variability has led researchers to investigate potential predictors of therapy response in order to determine which children and adolescents may benefit most from which type of upper limb therapy. Previous studies have already investigated potential neurological and behavioral predictors, however, sample sizes were often limited and only motor-based interventions were considered [32, 45, 46, 49–51]. Which predictors can be identified for somatosensory discrimination therapy remains to be investigated.

In summary, somatosensory function has shown to be crucial for upper limb motor function [1]. However, evidence underpinning the effectiveness of therapy approaches that specifically target somatosensory impairments is still scarce. Based on an earlier study, Sense for Kids has been proposed as a feasible and potentially effective therapy approach to improve bimanual performance and somatosensory function in children and adolescents with uCP. Yet, the effectiveness of this therapy to improve sensorimotor upper limb function when compared to upper limb motor therapy, its long-term effects, and potential predictors of therapy response still remain to be explored in an RCT.

## Objectives {7}

Therefore, this study protocol describes the design of an RCT comparing the efficacy of Sense for Kids therapy and dose-matched motor therapy to improve sensorimotor upper limb function in children and adolescents with uCP, who present with tactile impairments in the more impaired upper limb. The primary objective is to investigate if somatosensory discrimination therapy results in better bimanual performance at 6 months follow-up, compared to upper limb motor therapy. Secondly, we will investigate if somatosensory discrimination therapy is superior to improve somatosensory impairments. Lastly, we will explore the potential role of behavioral and neurological predictors of therapy response. The results of this study may aid in the selection of the most effective therapy intervention, specifically for children and adolescents with uCP who present with tactile impairments in the upper limb.

We hypothesize that somatosensory discrimination therapy will result in equal improvements in bimanual performance compared to upper limb motor therapy, immediately after the intervention period. However, we expect further improvements in bimanual performance during the follow-up period for participants who received somatosensory discrimination therapy because of the improved upper limb use in daily life, but not after upper limb motor therapy. We further hypothesize larger improvements in proprioceptive function and tactile perception, both immediately after the intervention and at follow-up, in participants who received somatosensory discrimination therapy. By considering the content and organization of the somatosensory discrimination therapy, we expect a higher therapy response for children and adolescents with worse somatosensory function and better attentional functioning at baseline. Lastly, we hypothesize that the occurrence of thalamic lesions is an important predictor of therapy response, because of the crucial role this brain structure has in the processing of somatosensory information that is transmitted from the periphery to the cortex [52].

## Trial design {8}

**Fig. 1:**
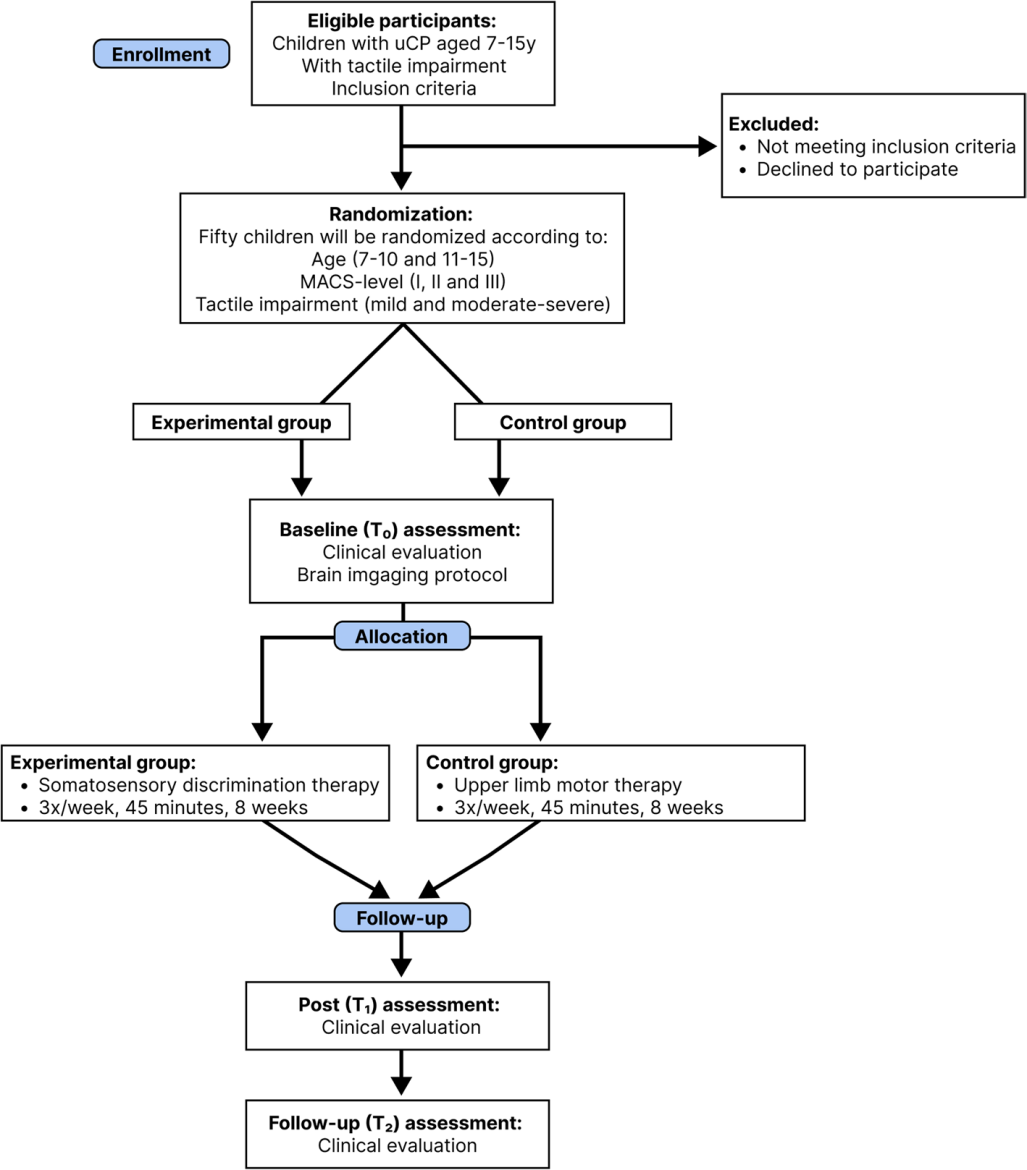
Flowchart of the described RCT based on the CONSORT guidelines [56]. Abbreviations: uCP = Unilateral cerebral palsy; MACS = Manual Ability Classification System; Baseline (T_0_) = Maximally 2 weeks before the start of the therapy; Post (T_1_) = Maximally 2 weeks after the therapy has ended; Follow-up (T_2_) = 6 months after the therapy has ended, within a time frame of 2 weeks

This study is a parallel group, evaluator-blinded, phase-II, single-center RCT [53]. Participants will be assigned to the intervention or active control group, with an equal (1:1) allocation ratio, based on stratified randomization. The study protocol is reported according to the SPIRIT (Standard Protocol Items: Recommendations for Interventional Trials) statement [54, 55]. Figure 1 gives a graphical overview of the described RCT. The study protocol, including the therapy intensity, practical organization of the therapy delivery, and duration of the assessments at each timepoint, was discussed with relevant stakeholders during online video-meetings to improve the feasibility of this RCT. The stakeholders that participated during these meetings were physiotherapists working in private practices across Flanders and children and adolescents with uCP and their parents.

## Methods: participants, interventions, and outcomes

### Study setting {9}

Children and adolescents with uCP will be recruited across Flanders (Belgium) via the Cerebral Palsy Reference Centre of the University Hospitals Leuven, private pediatric physiotherapists and special needs education schools.

### Eligibility criteria {10}

Participants will be selected based on the following inclusion criteria: (1) diagnosis of predominantly spastic uCP; (2) aged 7 to 15 years old at the time of baseline assessment; (3) sufficient cooperation to comprehend and complete the test and therapy procedures; (4) minimal ability to actively grasp and hold an object with the more impaired hand (Modified House Functional Classification System (mHFCS) ≥ 4 [57]); and (5) having a confirmed impairment in tactile registration and/or tactile perception, as objectified using a clinical assessment battery (see eligibility screening). The study will not include children and adolescents who (1) received botulinum toxin-A injections six months prior to the baseline assessment; (2) underwent upper limb surgery one year prior to the baseline assessment; and (3) are unable to communicate in Dutch. Eligibility of the participants will be confirmed by the researchers based on an eligibility screening, which is described below.

### Who will take informed consent? {26a}

Parents and potential participants will be informed about the study protocol by giving an in-person explanation, supported with written information documents. Afterwards, written informed consent to participate in the eligibility screening will be obtained from the parents or legal caregiver. Children and adolescents will be asked to additionally sign the informed assent form. When a child or adolescent meets the eligibility criteria based on the screening, an additional written informed consent and assent form to participate in the intervention study will be completed by the parents or legal caregiver and participant, respectively. The explanation of the study and informed consent procedure will be performed by one of the researchers.

### Additional consent provisions for collection and use of participant data and biological specimens {26b}

N/a—The collected participant data will not be used in ancillary studies.

## Interventions

### Explanation for the choice of comparators {6b}

Within this RCT, we aim to investigate if a therapy approach focusing on somatosensation has a superior effect to improve sensorimotor upper limb function in children and adolescents with uCP who present with tactile impairments in the upper limb, compared to a dose-matched motor therapy. A dose-matched active comparator was selected to eliminate differences in therapy intensity between both groups.

### Intervention description {11a}

Participants in both groups will receive three sessions of 45 minutes upper limb therapy per week for a period of eight weeks, resulting in a total therapy duration of 18 hours. All therapy sessions will be performed by individual physiotherapists or occupational therapists who have experience working with children with CP. The participant/therapist ratio will be 1:1 for both therapy groups.

#### Somatosensory discrimination therapy

Participants in the intervention group will receive the Sense for Kids therapy, which is a highly structured intervention program for the upper limb that aims to improve different aspects of somatosensory function [58]. More specifically, the therapy includes graded practice of three components of somatosensation (i*.*e., component-based therapy): tactile object recognition, texture discrimination, and body position sense. Furthermore, it contains the repetitive practice of self-chosen activities of daily living during which attention is designated to the somatosensory features necessary for successful task performance (i.e., occupation-based therapy). The self-chosen activities will be determined based on the results of the Canadian Occupational Performance Measure (COPM) [59], as described below. The same principles of perceptual learning as described in previous studies [36, 37, 41, 58] will be applied, consisting of active exploration without vision, calibration with the less impaired hand and with vision, feedback on performance and method of exploration, anticipation trials, repetition, and progression from easier to more difficult tasks. Each therapy session will consist of 30 minutes of component-based therapy, during which at least two different components of somatosensation will be practiced. These components will be selected based on the somatosensory profile of the child. The remaining 15 minutes consist of occupation-based therapy, during which one or two activities of daily living will be performed. To assure the quality of the therapy, all therapists who will perform the Sense for Kids therapy will follow an instructional course organized by the researchers. A therapy manual and all necessary therapy materials are developed based on the ones adapted by McLean et al. [41, 58] and will be provided to the therapists. The therapy manual contains detailed information regarding therapy delivery, points of attention, and methods of progression.

#### Upper limb motor therapy

Participants in the control group will receive an equal amount of intensive upper limb motor therapy. During each session, they will perform 30 minutes of unimanual motor tasks and 15 minutes of bimanual goal-directed training. For the unimanual motor tasks, a therapy manual will be provided by the researchers. This manual is developed in a similar manner as the one developed by Klingels et al. [44] and contains general information regarding therapy provision (i.e., progression/regression and number of repetitions). The manual also includes specific exercises grouped according to commonly reported upper limb motor problems: grasping and releasing, forearm supination, active wrist extension, thumb movements, and fine motor skills. Relevant exercises will be selected on an individual basis considering the upper limb function of the child or adolescent. This selection will be made by the researchers in collaboration with the individual therapist of the participant, taking into account measures of upper limb function performed at baseline and available in the medical record of the participant. During each session, participants will perform exercises targeting at least two different categories of upper limb problems. The bimanual goal-directed training includes repetitive whole-task practice of self-chosen activities of daily living, which will be determined based on the results of the COPM. During each session, one or two different activities of daily living will be practiced. Selected motor learning principles will be implemented throughout the upper limb motor therapy, including repetitive whole-task practice, progressive increases of task difficulty, provision of external feedback on task performance, and task specificity [19, 60, 61].

### Criteria for discontinuing or modifying allocated interventions {11b}

N/a—The participant may voluntarily withdraw consent to participate for any reason at any time. The participant’s request to withdraw from the study will always be respected without prejudice or further consequences.

### Strategies to improve adherence to interventions {11c}

To evaluate compliance, therapists from both groups will be asked to record the frequency and duration of the effective therapy time and the executed exercises/activities. After the first week of intervention and halfway through the intervention period, parents and therapists will be contacted by one of the researchers to discuss the feasibility of the therapy and the progress the child has made.

Furthermore, for the intervention group, adherence of the therapists to the Sense for Kids therapy protocol will be scored using a fidelity checklist that contains all fundamental elements of the therapy and relevant implications regarding therapy receipt (Additional file 1) [58]. The fidelity checklist was audited by five researchers and clinicians who have experience with the provision of Sense for Kids therapy. Modifications were made to improve clarity of the necessary elements, include essential elements of occupational-based therapy and remove assessment items to confine the forms application to individual and subsequent Sense for Kids therapy sessions. Fidelity of three randomly selected therapy sessions of each participant will be assessed based on a video recording of the therapy session. All video recordings will be scored by an independent reviewer who has experience with the Sense for Kids therapy but is not involved in the provision of therapy within the scope of this study. Each criterion will be scored on a 4-point Likert scale and a percentage score will be computed to objectify adherence. A percentage score of 80% or higher will be considered as sufficient adherence to the therapy protocol.

### Relevant concomitant care permitted or prohibited during the trial {11d}

In agreement with the treating physician and physiotherapist of the children and adolescents, conventional physiotherapy and occupational therapy for the upper limb will be replaced by the intervention within this study during the course of the 8-week intervention period. Other usual care, such as the use of splints and night orthoses or physiotherapy for the trunk and lower limbs, can be continued throughout the course of the study. During the follow-up period conventional physiotherapy and occupational therapy for the upper limb will be continued again. Information regarding the upper limb therapy received six months before therapy commencement and during the follow-up period will be collected based on a questionnaire completed by the parents and individual physiotherapist of the child or adolescent. When a participant receives botulinum toxin-A injections or upper limb surgery during the course of this study, the participant will be excluded and no additional data will be collected.

### Provisions for post-trial care {30}

N/a—After informed consent for the screening has been obtained until the last follow-up visit, all adverse events causally related to a study intervention will be reported according to the regulation of the Ethics Committee Research UZ/KU Leuven. Although adverse events and possible injuries are not expected due to the clinical nature of the study, participants are insured during their participation in all study-related interventions, including the assessments and therapy sessions, through the insurance taken by KU Leuven.

### Outcomes {12}

For the primary outcome and secondary outcomes, an overview of the specific outcome measures, analysis metrics, methods of aggregation, and timepoints of interest is shown in Table 1. Additional information regarding the outcome measures can be found under SPIRIT-item {18a}.

**Table 1:** Schematic overview of the specific outcome measures, analysis metrics, methods of aggregation and timepoints of interest for the primary outcome and secondary outcomes

		**Specific outcome measure**	**Analysis metric**	**Method of aggregation**	**Timepoints of interest**
**Primary outcome**					
	**Bimanual performance**	*(Adolescent) Assisting Hand Assessment (AHA/Ad-AHA)*	Change over time	Logit-based total score (0–100)	*T*_0_, *T*_2_
**Secondary outcomes**					
**Motor function**	**Bimanual performance**	*(Adolescent) Assisting Hand Assessment (AHA/Ad-AHA)*	Change over time	Logit-based total score (0–100)	*T*_0_, *T*_1_
		*Children’s Hand-use Experience Questionnaire (CHEQ)*		Logit-based total scores (0–100)	*T*_0_, *T*_1_, *T*_2_
	**Bimanual coordination**	*Kinarm exoskeleton: Ball-on-bar task*		Mean scores per level	
	**Bimanual coordination**	*Box opening task*		Mean scores over 5 trials	
	**Unimanual function**	*Tyneside pegboard test (TPT)*		Completion time in seconds	
	**Goal attainment**	*Goal Attainment Scaling (GAS)*		Classification based on criterion references into 6 categories (− 3–2)	
**Somatosensory function**	**Tactile registration**	*Semmes–Weinstein Monofilaments*		Classification based on threshold values into 5 categories (1–5)	
	**Tactile perception**	*Stereognosis assessment*		Number of correctly identified objects (0–6)	
		*Aesthesiometer*		Minimal distance in mm that can be correctly discriminated 5 consecutive times (3– > 10 mm)	
		*Tactile Discrimination Test*		Area under the curve (0–100)	
	**Proprioception**	*Movement sense*		Classification based on performance into 3 categories (0–2)	
		*Kinarm exoskeleton: Contralateral position matching task*		Mean scores across 24 trials	
		*Kinarm exoskeleton: Perceptual boundary task*		Slope and inflection point of the psychometric curve	
		*Kinarm exoskeleton: Indicating location task*		Number of correctly identified locations (0–12)	
		*ETH MIKE: Passive position sense task*		Mean absolute error across 11 trials	

*Abbreviations:* *mm* millimeters, *Baseline (T*_*0*_ *)* maximally 2 weeks before the start of the therapy, *Post (T*_*1*_ *)* maximally 2 weeks after the therapy has ended, *Follow-up (T*_*2*_ *)* 6 months after the therapy has ended, within a time frame of 2

### Participant timeline {13}

Table 2 gives a schematic overview of the study period and related aspects, including which outcome measure will be assessed at each timepoint.

**Table 2:**
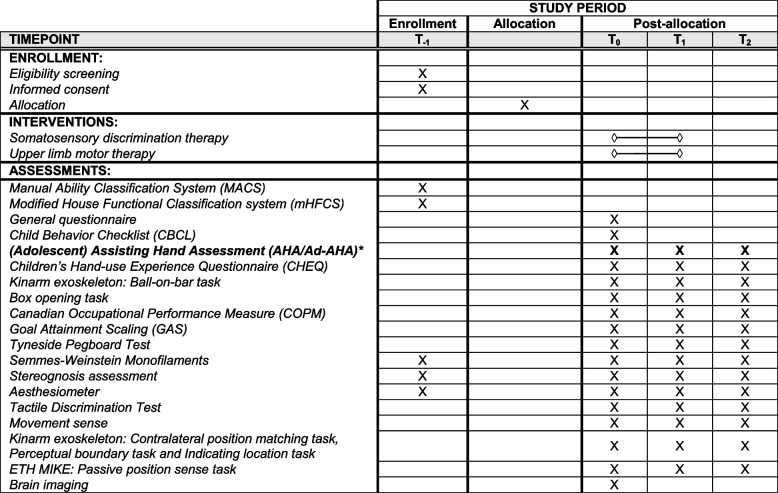
Schematic diagram of the enrollment, intervention and primary and secondary assessments, according to the SPIRIT statement [55]

*Abbreviations:* *Baseline (T*_*0*_ *)* maximally 2 weeks before the start of the therapy; *Post (T*_*1*_ *)* maximally 2 weeks after the therapy has ended; *Follow-up (T*_*2*_ *)* 6 months after the therapy has ended, within a time frame of 2 weeks

The primary outcome measure is presented in bold and indicated with an asterisk (*)

### Sample size {14}

The sample size calculation is based on the primary objective, which is defined as the retention of therapy effects on the primary outcome measure being bimanual performance measured with the Assisting Hand Assessment (AHA) at 6 months follow-up. The smallest detectable difference has been reported to be 5 AHA units [62]. Based on a previous intervention study in children with uCP [46], a standard deviation of 5.5 AHA units for improvements after intensive upper limb therapy was calculated by taking the average of the standard deviations between baseline and post-intervention and baseline and follow-up of both intensive therapy groups. The sample size estimate was calculated with G*Power for the following statistical test “Means: Difference between two independent means (two groups)” [63, 64]. With an estimated difference of 5 AHA units in the mean improvement between the intervention and control group and a standard deviation of 5.5 AHA units for both groups, an effect size of 0.9 was calculated. Based on an effect size of 0.9, an alpha level of 0.05 and a statistical power of 0.80, a sample size of at least 21 participants is needed in each group to detect a difference equal to or larger than the smallest detectable difference of 5 AHA units between groups. This number will be increased to 25 per group to account for inevitable dropouts and to maximize the study sample for prediction purposes.

### Recruitment {15}

Described above, under SPIRIT item {9}.

## Assignment of interventions: allocation

### Sequence generation {16a}

After enrollment and prior to the baseline assessment, participants will be assigned to the intervention or control group through randomization by minimization with an allocation ratio of 1:1. A minimization technique will be used to enhance homogeneity between both groups. To diminish allocation prediction, an additional random component of 80% probability will be implemented. The first participant, and in case the marginal totals are equal between both intervention groups, simple randomization will be performed. Participants will be stratified based on age (2 levels: < 11 years and ≥ 11 years), manual ability (3 MACS-levels: I, II, and III) and tactile impairment (2 levels: mild or moderate-severe – flowchart shown in Fig. 3).

### Concealment mechanism {16b}

Participants will be enrolled by one of the researchers, who will also perform the eligibility screening. Randomization will be performed by an independent person who is not involved in the selection procedure and will not have access to any additional clinical information about the participants [65]. All necessary participant information is provided to the independent person. In case of doubt or difficulties during randomization, a second independent person will be contacted to assist in resolving the difficulties.

### Implementation {16c}

Described above, under SPIRIT item {16b}.

## Assignment of interventions: Blinding

### Who will be blinded {17a}

The assessments of sensorimotor upper limb function at all three timepoints will be performed by a blinded researcher who was not involved in the conception of the study or the provision of therapy to the participants. Assessments that were video recorded (AHA and Goal Attainment Scaling (GAS)) will be scored afterwards by another evaluator who will be blind for group allocation and timepoint of assessment. Since the robotic measurements and three-dimensional motion analysis are fully automated, these assessments will be performed by a physiotherapist not blinded to group allocation. Participants and parents will be blinded to the study hypotheses. Lastly, the treating therapists will not be blinded to group allocation.

### Procedure for unblinding if needed {17b}

N/a—Because of the nature of the intervention and the related health risks, there are no occasions where unblinding is needed. Therefore, no specific procedure for unblinding was foreseen.

## Data collection and management

### Plans for assessment and collection of outcomes {18a}

An eligibility screening will be performed prior to randomization (*T*_−1_) to confirm eligibility of the child or adolescent based on the aforementioned in- and exclusion criteria and to objectify the stratification factors.

After randomization, assessments will be performed at *T*_0_ (baseline, within 2 weeks before the start of the therapy), *T*_1_ (post, within 2 weeks after the therapy has ended), and *T*_2_ (follow-up, 6 months after the intervention has ended within a 2-week time frame). Upper limb sensorimotor function will be comprehensively evaluated at each timepoint using clinical assessments with established measures of motor and somatosensory function and upper limb activities, as well as instrumented assessments with robotic measures and three-dimensional motion analysis. The assessments of sensorimotor upper limb function will be performed at the Faculty of Movement and Rehabilitation Sciences (KU Leuven, Belgium) and will be performed in the same order at all three timepoints. Furthermore, brain imaging will be performed at baseline at the University Hospitals Leuven (Belgium). All assessments will be performed by the same researchers, who have experience working with children with uCP.

**Fig. 2:**
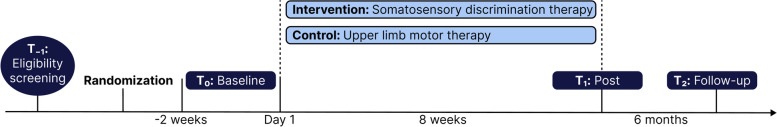
Illustration of the study design and assessment timepoints. Abbreviations: Baseline (*T*_0_) = maximally 2 weeks before the start of the therapy; Post (*T*_1_) = maximally 2 weeks after the therapy has ended; Follow-up (*T*_2_) = 6 months after the therapy has ended, within a time frame of 2 weeks

A summary of the assessment timepoints is presented in Fig. 2.

#### Eligibility screening

During the eligibility screening, the participants will be classified based on their upper limb function according to the MACS and the mHFCS. The MACS is a five-level scale indicating the ability of children and adolescents with CP to handle objects during daily life activities (Level I = “Handles objects easily and successfully”; Level V = “Does not handle objects and has severely limited ability to perform even simple actions”) [66, 67]. The mHFCS is used to describe the role of the hands during the performance of bimanual activities. The nine levels of the mHFCS range from a hand that is not used at all (grade 0) to completely independent and spontaneous hand use without reference to the other hand (grade 8) [57]. An overview of all classification levels of both scales is presented in Additional file 2.

**Fig. 3:**
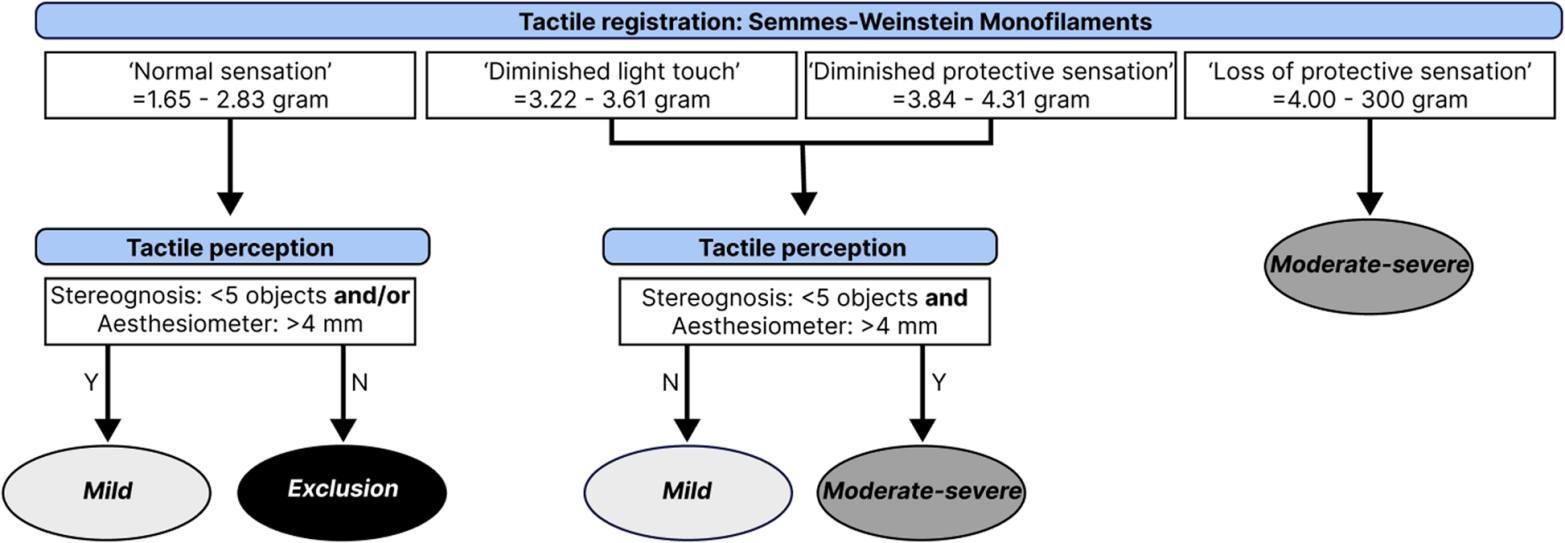
Flowchart for categorization of tactile impairments, used for stratification. Abbreviations: Y = yes; N = no; mm = millimeters

To confirm the presence of a tactile impairment in the more impaired hand, a short evaluation of tactile registration (Semmes–Weinstein Monofilaments) and tactile perception (stereognosis and aesthesiometer) will be performed. During the eligibility screening, these assessments will only be performed for the more impaired hand. Additional information regarding the administration of these assessments can be found below, under the section “Somatosensory function”. A graphical overview of the cut-off values used for each assessment to confirm the presence of a tactile impairment is presented in Fig. 3.

The assessments for this eligibility screening were carefully selected to minimize the duration as much as possible, so that the screening can be performed by one of the researchers during the routine follow-ups in the Cerebral Palsy Reference Centre of the University Hospitals Leuven. Therefore, the researchers have decided to focus on the tactile impairments, which have shown to be more prevalent based on clinical assessments [23], and to not perform a screening of proprioceptive function.

#### Clinical evaluation

##### Participant characteristics

General participant characteristics, including date of birth, sex, comorbidities, more impaired side, hand preference, and current upper limb therapy, will be collected at baseline based on a parental questionnaire and by consulting the medical file and/or individual physiotherapist of the participants. Parents will be further asked to complete the Dutch version of the Child Behavior Checklist for children and adolescents aged 6 to 18 years old (CBCL 6–18, Dutch parental version) [68]. This questionnaire consists of 120 items describing internalizing, externalizing, and total behavioral problems in school-aged children and adolescents. Eight syndrome scales are calculated based on the results: (1) anxious/depressed behavior, (2) withdrawn/depressed behavior, (3) somatic complaints, (4) social problems, (5) thought problems, (6) attention problems, (7) rule-breaking behavior, and (8) aggressive behavior. For the scope of this study, the results of the attention problems subscale will be considered as a potential predictor of therapy response.

##### Primary outcome measure

- Assisting Hand Assessment, Adolescent Assisting Hand Assessment

The AHA and Adolescent Assisting Hand Assessment (Ad-AHA) evaluate the spontaneous use of the more impaired upper limb during bimanual activities [69, 70]. A semi-structured play session with standardized toys and materials that require the use of both hands is performed and video recorded. Subsequently, 20 items are scored on a 4-point Likert scale ranging from 1 (‘does not do’) to 4 (‘effective use’) and a logit-based total score in AHA units is calculated. The AHA and Ad-AHA have shown to be reliable and valid assessments for children and adolescents with uCP [71–74]. The AHA or Ad-AHA will be evaluated at each timepoint, depending on the age of the participant. The AHA will be the primary outcome measure for this intervention study, because the ultimate aim is to improve the use of the impaired upper limb during daily life activities.

##### Secondary outcome measures

**Somatosensory function**

- Clinical assessments

Somatosensory function will be measured clinically at all timepoints. These assessments comprise standardized measures for tactile registration, tactile perception, and proprioception. In all clinical assessments, except for the one of stereognosis, the less impaired upper limb is assessed first followed by the more impaired upper limb. To determine threshold values for tactile registration, the Semmes–Weinstein Monofilaments will be used. This test consists of a kit of 20 nylon monofilaments (Jamar® Monofilaments, Sammons Preston, Rolyan, Bolingbrook, IL, United States) [12, 75]. The score is the lowest filament at which the participant was able to correctly identify three consecutive touches. The assessment starts with the monofilament that represents the lower border of normal tactile registration (0.07 g). Afterwards, difficulty is increased (i.e., lower filament) or decreased (i.e., higher filament) depending on the performance. Tactile registration will be classified as normal (0.008–0.07 g), diminished light touch (0.16–0.4 g), diminished protective sensation (0.6–2 g), and loss of protective sensation (4.19–300 g). Next, three different components of tactile perception will be assessed: (1) stereognosis or tactile object recognition, (2) two-point discrimination, and (3) texture discrimination. First, stereognosis will be assessed through the tactile identification of familiar objects. This assessment will be performed as described by Klingels et al. [76]. Participants are asked to identify six out of twelve randomly selected objects, of which three are matched in pairs similar in size and shape (pencil/pen, coins/button, paperclip/safety pin) and three clearly different objects (marble, spoon, comb, ball, clothespin, key). The score is the number of correctly identified objects. Second, two-point discrimination will be evaluated using an Aesthesiometer which is placed longitudinally and perpendicularly on the fingertip of the index, as described by Klingels et al. [76]. The test score is the minimal distance that the participant could still discriminate as two discrete points in five consecutive trials. The assessment starts with a distance of 4 mm that is progressively decreased or increased depending on the participant’s performance. A distance higher than 4 mm is considered impaired [77]. Third, the Tactile Discrimination Test will be used to objectify texture discrimination [78, 79]. During this test, participants are guided to feel several triplets of textures with their index finger and are asked to identify which texture out of each set of three is different. The score is calculated based on the difference in texture that the participant is still able to correctly differentiate. Lastly, passive movement sense of the index finger will be evaluated as a measure of proprioception. During this assessment, the metacarpophalangeal joint of the index finger is moved over a small amplitude by the assessor and the participant is asked to indicate if the movement was perceived. If the movement was not perceived, the same procedure is repeated with a larger movement amplitude. Movement sense will be classified as normal (score 2—small amplitude perceived in all three attempts), impaired (score 1—only larger amplitude perceived), or absent (score 0—no movement perceived) [76].

- Kinarm Exoskeleton: Contralateral position matching task, Perceptual boundary task and Indicating location task

Proprioception of the shoulder and elbow joints will be assessed simultaneously using the Kinarm Exoskeleton robotic device (Kinarm, BKIN Technologies Ltd, Kingston, Canada). Participants are seated on a height-adjustable chair with both upper limbs positioned in individually calibrated arm supports, allowing for upper limb movements in the horizontal plane. A virtual reality screen is positioned above the upper limbs to display task visuals and to occlude direct vision of the upper limbs. To objectify proprioception, three different tasks will be performed: (1) the contralateral position matching task, (2) the perceptual boundary task, and (3) the indicating location task. All tasks are first performed with the less impaired upper limb, followed by the more impaired upper limb. First, the contralateral position matching task is a valid measure of static position sense during which the robot passively moves the upper limb that is being assessed to one out of four spatial targets, each separated by 12 cm [80, 81]. Afterwards, the participant is instructed to actively mirror-match this position with the contralateral upper limb. Each participant performs 24 trials, resulting in four parameters describing position sense (i.e., absolute error, variability, contraction/expansion and systematic shift). The second task, the perceptual boundary task, is an adapted version of the task described by Vandevoorde et al. [82]. During this task, a passive upper limb reaching movement with an angular deviation relative to a straight line is performed by the robotic device. The robot then passively returns the upper limb to the starting position. Afterwards, participants are asked to indicate whether the performed movement deviated to the left or right compared to a straight line. The angular deviation applied on the next trial depends on the performance and is determined via a parameter estimation by sequential testing procedure, in order to estimate how accurately each participant can discriminate angular deviations. A more detailed description of the procedure to calculate the final parameter can be found elsewhere [82]. Some minor adaptations compared to the protocol described by Vandevoorde et al. were made to increase applicability in children and adolescents with uCP. Colored symbols were added on the left and right side of the virtual screen to assist in describing the direction of the deviation (i.e., left/yellow duck and right/green car). Next, a straight reference line was added on the screen to improve task comprehension. Lastly, the movement was made passive to avoid interference of motor impairments that might be present in the upper limb. In the third proprioceptive task (indicating location task), the robot passively moves the upper limb to one out of four prespecified locations. The participant is then asked to indicate the perceived position of the tip of their index finger. Each potential location is marked by a colored circle containing a white figure to assist in describing the location (i.e., apple/green, tree/red, flower/yellow, house/blue), with at least 5 cm of distance between neighboring locations. Each participant will perform a total of 12 trials. The final score is the number of trials during which the participant correctly identified the location of the hand. The three tasks will be performed at all timepoints.

- ETH MIKE: Passive position sense task

The ETH MIKE (Motor Impairment and Kinesthetic Evaluation) robot (Rehabilitation engineering laboratory, ETH Zürich, Zürich, Switzerland) will be used to measure proprioception of the metacarpophalangeal joint of the index finger during a passive position sense task. The ETH MIKE is a one degree of freedom end-effector that can accurately measure positions and movements. Participants are seated in front of the device with their hand grasping a handle and their index finger attached to the end-effector using Velcro straps. The center of rotation is aligned with the metacarpophalangeal joint of the index finger. A tablet computer is positioned above the participant’s hand to display the task visuals and to block direct vision of the hand. During the passive position sense task, the participant’s index finger is passively moved from a starting position of 0° flexion to one out of 11 predefined positions, ranging from 10 to 30° flexion. The participant is then asked to indicate their perceived finger position on the tablet screen located above the hand. This results in two parameters of interest: the mean absolute error between the actual and the indicated position across the 11 trials and the mean variability over these trials. This task has shown to be valid and reliable in children and adolescents with uCP and will be performed at all timepoints [83]. More in-depth details regarding the ETH MIKE can be found elsewhere [83–85].

##### Motor function

- Children’s Hand-use Experience Questionnaire

The Children’s Hand-use Experience Questionnaire (CHEQ) is an online questionnaire that captures the child’s experience of using the more impaired hand during 29 daily bimanual activities (available online at: http://www.cheq.se/questionnaire) [86]. This questionnaire will be completed by the parents or legal caregiver. For each bimanual activity, the parents are asked to indicate if the child or adolescent uses one or both hands to perform the activity or if assistance is needed. Followed by three sub-questions that are scored on a 4-point Likert scale, indicating the (1) efficacy of the more impaired hand, (2) time needed to perform the activity compared to peers, and (3) if the child feels bothered by the more impaired hand when performing the activity. The CHEQ has shown to be reliable and valid for the use in children and adolescents with uCP [87]. This questionnaire will be completed at each timepoint.

- Kinarm Exoskeleton: Ball-on-bar task

The standardized ball-on-bar task will be performed on the Kinarm Exoskeleton to quantify bimanual coordination. The fingertips of the participant are connected using a virtual bar of 20 cm on which a ball is positioned. The participants are instructed to move the ball to circular targets, while keeping it balanced on the bar. The task has two difficulty levels; in the first level, the ball is fixed to the bar, while in the second level, the ball moves when the bar is being tilted. A more detailed description of the set-up and task can be found elsewhere [81, 88]. Parameters of interest are determined based on previous research in children and adolescents with uCP [89] and include bar tilt standard deviation, hand path length bias, reaction time difference, and hand speed difference. This task will be performed at every timepoint.

- Box opening task

To evaluate temporal aspects of bimanual coordination, three-dimensional motion analysis will be performed during a bimanual box opening task (Oxford Brookes University, Oxford, UK). Participants are requested to open the lid of a transparent box with one hand and press a button inside the box using their contralateral hand at a self-selected pace. The task will be performed under two conditions: opening the box with (1) the more impaired hand and (2) the less impaired hand. Three-dimensional motion-tracking sensors (Polhemus, Colchester, Vermont, USA) are placed on the dorsal side of the third metacarpal bone. More detailed information regarding this assessment and data analysis is reported elsewhere [90, 91]. Parameters of interest are total movement time and goal synchronization. Again, these parameters are determined based on an earlier study in children and adolescents with uCP and typically developing peers [92]. Participants will be asked to perform this task at every timepoint.

- Canadian Occupational Performance Measure

The COPM is a measure of the participant’s self-perceived occupational performance and satisfaction in functional goals within the domains of self-care, leisure, and productivity [59]. The COPM is a standardized test in which maximally five functional goals are identified by the parents or legal caregiver and/or the participant during a semi-structured interview [93]. The performance and satisfaction of each functional goal is rated on a 10-point ordinal scale, where 1 = “not able to do it all”/ “not satisfied at all” and 10 = “able to do it extremely well”/ “extremely satisfied”. The validity, reliability, and responsiveness of the COPM have been shown previously [94, 95]. The parents and participants will be asked to rank the identified goals from most important to least important. The three goals that are identified as most important will be practiced during the therapy sessions. Mean performance and satisfaction scores for the functional goals that were practiced and the ones that were not practiced during therapy will be analyzed separately to investigate therapy effects.

- Goal Attainment Scaling

Therapy effects on a functional level will be further measured using GAS. GAS is a valid and reliable, criterion-referenced measure that is used to quantify achievement of functional goals on a 6-point scale, ranging from − 3 to 2 [96–98]. The participant’s baseline performance is represented by a score of − 2. Improvements in functional goal performance correspond to scores ranging from − 1 to + 2, with score 0 being the expected outcome. Deterioration in functional goal performance results in a score of − 3. For each participant, the most and least important functional goal identified by the COPM will be translated into a GAS, resulting in a GAS for one practiced and one unpracticed functional goal. Performance of these functional goals will be video recorded and scored at each timepoint.

- Tyneside Pegboard Test

The Tyneside Pegboard Test (Newcastle University, Newcastle upon Tyne, UK) will be used to assess unimanual dexterity. Validity and reliability of this test in children and adolescents with uCP have been shown [25, 99]. Participants are asked to transfer nine medium-sized pegs from one board to an adjacent one as quickly as possible, using first the less impaired and then the more impaired hand. Completion time is electronically collected and results are outputted using a custom-written software. This task will be performed at all three timepoints.

#### Brain imaging

A brain magnetic resonance imaging (MRI) scan will be performed at baseline on a 3.0 Tesla MRI scanner with a 32-channel head coil (Hercules, Philips Medical Systems, Best, The Netherlands). The imaging protocol consists of structural MRI for the anatomical characterization of the brain lesion (i.e., lesion timing, location and extent) and diffusion-weighted imaging (dMRI) to investigate white matter structural connectivity. For children up to the age of 10 years old and in case of anxiety for the MRI assessment, a familiarization protocol will be performed before the actual scan to introduce important characteristics of the assessment (e.g., noise, small space, lying still, headphones for communication) [100]. Furthermore, all participants will be allowed to watch a video of their choice during the entire scan.

##### Structural MRI

High-resolution T1-weighted images will be acquired with the following parameters: echo time = 4.2 ms, repetition time = 9.1 ms, inversion time = 760.3 ms, field of view = 256 FH × 180 RL × 242 AP mm^3^, voxel size = 0.9 × 0.9 × 0.9 mm^3^, and 3D turbo spin echo. In addition, T2-weighted fluid-attenuated inversion recovery images will be acquired with the following parameters: echo time = 283 ms, repetition time = 4800 ms, inversion time = 1650 ms, field of view = 250 FH × 200 RL × 250 AP mm^3^, and voxel size = 1.0 × 1.0 × 1.0 mm^3^. Lastly, also T2-weighted images will be collected with the following parameters: echo time = 280 ms, repetition time = 3000 ms, inversion time = 548 ms, field of view = 256 FH × 198 RL × 256 AP mm^3^, and voxel size = 1 × 1 × 1 mm^3^.

The MRI classification system (MRICS) will be applied to the anatomical images in order to classify the brain lesions according to the timing of the lesion and the predominant lesion pattern, resulting in five main groups: (1) maldevelopments, (2) predominant white matter injury, (3) predominant gray matter injury, (4) miscellaneous, and (5) normal findings [101]. Next, lesion location and extent will be evaluated in more detail using the semi-quantitative MRI (sqMRI) scale developed by Fiori et al. [102, 103]. In short, the sqMRI scale consists of a graphical black and white template on which the lesion is drawn. This template is then used to quantitatively analyze the lesion characteristics, resulting in different global and subscores that separately assess both hemispheres, different brain regions and depths. More detailed information regarding the MRICS and sqMRI can be found in earlier publications [101, 102].

##### Diffusion-weighted images.

Multi-shell diffusion-weighted images will also be acquired using a 2D single-shot spin echo sequence with the following specifications: slice thickness = 2.3 mm, echo time = 93 ms, repetition time = 3765 ms, anterior–posterior phase encoding direction, b-values = 0/1000/25000 s/mm2 with 3/50/74 uniformly distributed gradient direction respectively, in-plane parallel acceleration factor = 1.5 [104], field of view = 136 FH × 240 RL × 240 AP mm3, voxel size = 2.2 × 2.2 × 2.2 mm3, acquisition time = 8 min. There were 124 uniformly distributed diffusion directions. dMRI will be pre-processed using an MRTrix-based pipeline [105]. Constraint spherical deconvolution will be applied to delineate the motor and somatosensory tracts for both hemispheres (e.g., corticospinal tract, thalamic radiations, medial lemniscus) [106, 107].

### Plans to promote participant retention and complete follow-up {18b}

To avoid dropouts due to practical considerations, the scheduling of therapy and assessment sessions will be adapted to the participant’s preferences where possible. Furthermore, one contact person will be appointed to coordinate the scheduling of therapy and assessments and to resolve any questions. Lastly, parents will be contacted after the first week of therapy and halfway the intervention period to discuss feasibility of the therapy and the progress the child has made. In case of deviations from the study protocol (e.g., missed therapy sessions due to illness), the assessments will still be scheduled as described above and the specific deviation will be noted.

### Data management {19}

All data, including assessment results, video recordings, and activity logs, will be digitalized and stored pseudonymized on a secured university network and/or on the REDCap platform. To ensure data quality, double data entry will be performed by two independent researchers. Any discrepancies will be checked and adjusted by a third researcher using the Data Comparison Tool of the REDCap platform. Moreover, range checks will be built-in on REDCap for all quantitative outcome measures. Anomalies and data outliers will be inspected and adapted when appropriate before data analysis. The final trial dataset will be accessible on REDCap to the day-to-day management team.

### Confidentiality {27}

An individual, study-specific identifier will be assigned to all participants upon enrollment to guarantee confidentiality. Next, a participant identification record will be generated, containing personal information, and contact details. This record will be stored separately and access will be restricted to two researchers, who will also be responsible for the pseudonymization. No identifiable or personal data will be published or made available to other researchers. The de-identified dataset will be made publicly available upon publication of the study results via KU Leuven Research Data Repository.

### Plans for collection, laboratory evaluation and storage of biological specimens for genetic or molecular analysis in this trial/future use {33}

N/a—No biological samples for genetic or molecular analysis will be collected.

## Statistical methods

### Statistical methods for primary and secondary outcomes {20a}

All observations will be summarized as mean and standard deviation or median and interquartile range, according to the nature of the data. Baseline comparability between groups will be visually checked based on descriptive statistics. As the participants will be randomized to the intervention or control group, statistical testing of baseline differences will not be performed [108]. To study the time effects of the intervention and to allow for comparison between both groups, linear or generalized mixed models will be applied for the primary and secondary outcome measures. By using random effects, such models correct for the relation among repeated observations within subjects. An additional benefit of these models is that they provide a valid inference for missing values that are (completely) at random [109]. In case the data of an outcome measure is not normally distributed based on the Shapiro–Wilk test and/or visual inspection of the data distribution, transformations will be applied. The main endpoint will be to investigate if there is a difference in evolution of the AHA score over time between both groups. As such, interactions between therapy and time will be analyzed to test for differences in improvements over time between both groups (time*group interaction effect). When a significant interaction is identified, time trends will be tested in both therapy groups separately (time-effect). When the interaction is not significant, the main effect of time will be explored. Effect sizes for the full models will be further calculated using the Cohen’s partial η^2^ formula [partial η^2^ = (F × df_between_)/((F × df_between_) + df_within_)] [110]. Significant time trends will be further investigated with pairwise post hoc tests to compare individual time points (*T*_0_–*T*_1_, *T*_1_–*T*_2_, *T*_0_–*T*_2_). For the model of the primary outcome measure, the post hoc analysis will be Bonferroni corrected and the pairwise post hoc test for *T*_0_–*T*_2_ will be the primary outcome. For the models of secondary outcome measures, a false discovery rate with an adjusted *p*-value of 0.05 will be implemented. Effect sizes of these comparisons will be calculated using Cohen’s d formula [111]. For the second research objective, we will investigate predictive factors of therapy response. Both behavioral and neurological characteristics, i.e., somatosensory function at baseline, attentional functioning, and thalamic volume, will be included as covariates in the model for the primary outcome measure. All statistical analyses will be performed in SPSS Statistics for Windows. Level of significance will be set at 0.05, two-sided. When needed, adapted statistical techniques will be applied in close collaboration with Leuven Biostatistics and statistical Bioinformatics Centre (L-BioStat).

### Interim analyses {21b}

No interim analyses are planned at this moment. However, the trial steering group can request interim analysis at any time. In this case, the analysis will be performed by one of the main researchers and results will be discussed with the trial steering group.

### Methods for additional analyses (e.g., subgroup analyses) {20b}

Described above, under SPIRIT item {20a}.

### Methods in analysis to handle protocol non-adherence and any statistical methods to handle missing data {20c}

Described above, under SPIRIT item {20a}.

### Plans to give access to the full protocol, participant-level data, and statistical code {31c}

N/a—The full trial protocol will be available at ClinicalTrials.gov and upon publication of this protocol paper. The de-identified dataset will be made publicly available upon publication of the study results via KU Leuven Research Data Repository.

## Oversight and monitoring

### Composition of the coordinating center and trial steering committee {5d}

Different working groups have been established to organize the design, execution, and follow-up of this intervention study. Table 3 provides an overview of the composition, roles, and responsibilities and meeting frequency of the involved working groups.

**Table 3:** Schematic overview of the composition, roles and responsibilities, and meeting frequency of the working groups providing trial support

	**Composition**	**Roles and responsibilities**	**Meeting frequency**
**Trial steering group**	Day-to-day management team, child neurologist, child orthopedic surgeon, experts in the field of (pediatric) rehabilitation	- Agreement on final protocol - Reviewing study progress and if necessary agreeing on changes to the protocol - Assistance in patient recruitment - Data analysis and interpretation - Reporting of trial results to relevant parties - Advice on publicity and presentation of all aspects of the trial	Three times a year
**Day-to-day management team**	Principle investigator and main researchers	- Study planning and coordination (e.g., communication between involved parties) - Budget administration - Initial preparation and revisions of the study protocol and related documents - Organization of meetings with the trial steering group - Clinical trial registration (e.g., ClinicalTrials.gov) - Trial conduct (e.g., scheduling therapy sessions, performance of eligibility screening, study logistics) - Recording and reporting of adverse events - Data quality assurance - Provision of annual progress report to the Ethics Committee Research UZ/KU Leuven	Monthly meetings
**Principal investigator**	Senior professor at KU Leuven	- Supervise trial conduct - Protect rights, safety and well-being of participants - Responsible for data storage and preservation	/
**Data management team**	Main researchers	- Build trial specific database on REDCap platform - Data entry and verification	Monthly meetings
**Stakeholders**	Physiotherapists working in private practices across Flanders and children and adolescents with unilateral cerebral palsy and their parents	- Advice on study protocol (e.g., therapy intensity, duration of therapy and assessment sessions) - Discussions on strengths/weaknesses/opportunities/threats of this study	Occasional video-meetings

### Composition of the data monitoring committee, its role and reporting structure {21a}

N/a—No data monitoring committee will be composed for this trial because of the activity-based character of the therapy and assessments, which have shown to be safe in earlier studies.

### Adverse event reporting and harms {22}

Information regarding adverse events will be collected from the participants and their parents through in-person conversations during each participant contact. According to the regulations of the Ethics Committee Research UZ/KU Leuven, all adverse events will be evaluated by the researchers as to seriousness, severity, and causality to the therapy or assessments. Depending on the severity of the adverse event, appropriate follow-up and reporting of these events will be organized by the researchers, and in consultation with the involved medical doctors.

### Frequency and plans for auditing trial conduct {23}

First, to comply with the sponsor responsibilities related to study monitoring, UZ/KU Leuven has appointed the Clinical Trial Center (CTC), who is not involved in the execution of clinical studies, to perform audits on UZ/KU Leuven-sponsored studies. These audits are organized following a risk-based approach. Secondly, general trial conduct will be evaluated by the day-to-day management team. These evaluations include source data verification, follow-up of issues during data collection, and validation of missing data. The day-to-day management team will further submit a yearly progress report to the Ethics Committee Research UZ/KU Leuven, according to the regulation of good clinical practice (ICH-GCP). Lastly, the researchers will permit study-related monitoring, audits, ethical committee review, and regulatory inspection by any competent authority, providing direct access to all related source data and/or documents.

### Plans for communicating important protocol amendments to relevant parties (e.g., trial participants, ethical committees) {25}

If important protocol modifications are required, a valid and substantial amendment will be prepared by the principal investigator, upon consultation of the trial steering group. This amendment will be submitted to the Ethics Committee Research UZ/KU Leuven for approval. When ethical approval is obtained, the protocol registration at ClinicalTrials.gov will be updated using tracked modifications. Any changes compared to the initial protocol will further be pointed out in scientific publications of the study results.

### Dissemination plans {31a}

The results of this study will be disseminated by peer-reviewed journal publications as well as active participation at international conferences. Authorship of publications will be determined in accordance with the guidelines of KU Leuven and in accordance with the requirements of the respective (medical) journal. We will further participate in activities and events that focus on science communication to a non-expert audience, including parents, children and adolescents with and without disabilities and clinicians.

## Discussion

This study aims to investigate the efficacy of somatosensory discrimination therapy and dose-matched motor therapy to improve sensorimotor upper limb function in children and adolescents with uCP who present with tactile impairments. We will further investigate the potential role of different behavioral and neurological predictors of therapy response. Our main hypothesis is that somatosensory discrimination therapy will result in improved bimanual performance at follow-up, compared to dose-matched motor therapy.

A comparable trial protocol has been published in 2018 by McLean and colleagues [58]. However, due to difficulties with recruitment during the COVID19 period, earlier discontinuation of this trial was necessary. As only six children with uCP had completed the intervention, preliminary analysis of the trial results was impossible. Nevertheless, their earlier feasibility study has shown that Sense for Kids is a feasible and engaging therapy for children and adolescents with uCP [41, 42].

Despite the fact that somatosensory function has shown to be critical to coordinate and finetune movements of each hand separately to a skilled bimanual performance [1], therapy programs that specifically target the somatosensory impairments are scarce. To the best of our knowledge, this is the first RCT comparing the effectiveness of an intervention approach specifically designated to address somatosensory impairments to a dose-matched motor therapy in children and adolescents with uCP that present with tactile impairments in the upper limb. Although there is favorable evidence for this therapy concept to improve upper limb function in adult stroke patients [28, 33, 34]. Sensorimotor upper limb function will be comprehensively evaluated using clinical assessments that have shown to be valid and reliable in children and adolescents with uCP, as well as based on robotic evaluations and instrumented three-dimensional motion analysis.

Gaining insight in how somatosensory discrimination therapy impacts upper limb function might be important to improve current upper limb therapies specifically for children and adolescents with uCP who present with tactile impairments. If targeting somatosensory impairments in the upper limb could lead to a breakdown of the vicious circle of somatosensory impairments, reduced use of the impaired upper limb during bimanual activities, and reduced developmental experience, this therapy could result in improved functional independence and quality of life. By investigating potential predictors of therapy response, the results of this study may aid in further individualization of upper limb therapy.

## Trial status

Recruitment started on August 8, 2023. The anticipated end-date of recruitment is 30 June 2027 or when the total number of 50 participants is reached. The trial will be conducted according to the protocol (version 5.2 – 19 January 2024) for which ethical approval was obtained.

### Supplementary Information

**Additional file 1.** Therapy Fidelity Checklist for the somatosensory discrimination therapy.

**Additional file 2.** Description of the different categories of upper limb function classifications. Manual Ability Classification System.

## Data Availability

The de-identified dataset will be made publicly available upon publication of the study results via KU Leuven Research Data Repository.

## Abbreviations

CP: Cerebral palsy
uCP: Unilateral cerebral palsy
RCT: Randomized controlled trial
mHFCS: Modified House Functional Classification System
COPM: Canadian Occupational Performance Measure
AHA: Assisting Hand Assessment
MACS: Manual Ability Classification System
GAS: Goal Attainment Scaling
CBCL: Child Behavior Checklist
Ad-AHA: Adolescent Assisting Hand Assessment
CHEQ: Children’s Hand-use Experience Questionnaire
ETH MIKE: ETH Motor Impairment and Kinesthetic Evaluation
MRI: Magnetic resonance imaging
dMRI: Diffusion-weighted imaging
MRICS: MRI classification system
sqMRI: Semi-quantitative MRI scale
