# Supplementary material for: Does somatosensory discrimination therapy alter sensorimotor upper limb function differently compared to motor therapy in children and adolescents with unilateral cerebral palsy: study protocol for a randomized controlled trial
Source: Trials. 2024 Feb 26;25:147. doi: 10.1186/s13063-024-07967-4 (PMC10895830; doi:10.1186/s13063-024-07967-4)
Supplement: Supplementary file 1 — Additional file 1. Therapy Fidelity Checklist for the somatosensory discrimination therapy. [file 13063_2024_7967_MOESM1_ESM.pdf]

Additional file 1: Therapy Fidelity Checklist for the somatosensory discrimination therapy.

Somatosensory discrimination therapy (Sense © for Kids) **Fidelity Checklist**

**Therapist:**	
**Fidelity Assessor:**	
**Project ID:**	
**Date of Assessment:**	
**Therapy session code:**	
**Score:**	**0**

1: Never (0- 25%); 2: occasionally/ most often not (26- 50%); 3: most often 51- 75%; 4: always (76- 100%)

			Likert Scale				
		**Delivery of treatment**	**1**	**2**	**3**	**4**	**Observations**
**Subcomponent training**							
	1	The therapist addresses two of the four domains in each treatment session (body position sense, tactile discrimination, haptic object recognition and occupation training).					
Active exploration	2	The therapist facilitates the child's systematic exploration of the item without vision.					
	3	The therapist presents the stimulus item using optimal exploratory procedures as outlined in the training document.					
	4	The therapist facilitates the child's description of the presented somatosensory stimuli.					
	5	The therapist responds appropriately by guiding calibration or providing anticipation activity.					
	6	Therapist provides knowledge of results feedback.					
Calibration	7	During calibration the therapist reveals the stimuli for visual calibration and presents the stimuli to the less affected hand.					
	8	The therapist facilitates the child's exploration of the stimuli with their less affected hand, then with their more impaired hand and facilitates the use of imagination to "match" the sensation.					
	9	The therapist provides predominantly knowledge of performance feedback (method of exploration).					
	10	The therapist monitors the child's understanding of the purpose of the use of imagination.					
Anticipation	11	The therapist facilitates presentation of known stimuli to the child.					
	12	The therapist provides predominantly knowledge of results feedback.					
	13	The therapist facilitates repetition of the activity until the child achieves 3 out of 4 correct.					
	14	The therapist demonstrates the ability to move between anticipation and calibration based on the child's response.					
Repeat and progress	15	The therapist progresses to the next task when the child achieves 3 out of 4 correct.					
**Occupational task practice**	16	Upon completion of the subcomponent training, occupational goal is trained with emphasis on sensory stimuli.					
	17	Occupational task practice involves the child practicing one or more of their self-selected goals with the therapist cueing them to notice the obvious somatosensory attributes of the task and materials necessary for succesful task completion.					
	18	The therapist cues the child to notice the **feel of item/object properties** in the hand during task practice.					
	19	The therapist cues the child to notice the feel of item/object properties in **correct placement for best use** in the hand during task practice.					
	20	The therapist cues the child to notice the feel of their **hand/upper limb position** during task practice and cues to match other arm emphasising calibration as outlined in item 8.					
	21	The therapist cues the child to notice the feel of **resistance of the item/object during use** in task practice (such as the hand-feel of a fork used with different textures of food, resistance buttoning through small button holes) .					
**Therapy set-up**	22	The therapist creates an environment supporting focussed attention and learning (distractions are removed…).					
	23	The therapist responds to the child's behaviour and modifies therapy as required.					
	24	The child is actively engaged in the therapy session and tasks.					
**Receipt of treatment**	34	The child is able to use the calibration strategy and subsequently demonstrates improvement.					
	35	Child demonstrates understanding of the task requirements and what is expected.					
	36	Child recognises achievement of their goals.					
	37	Child is enjoying the session.					
	38	The child is actively utilising the information provided by the therapist to modify performance.					
	**TOTAL**		**0**	**0**	**0**	**0**<br>0	
