# Supplementary material for: Does somatosensory discrimination therapy alter sensorimotor upper limb function differently compared to motor therapy in children and adolescents with unilateral cerebral palsy: study protocol for a randomized controlled trial
Source: Trials. 2024 Feb 26;25:147. doi: 10.1186/s13063-024-07967-4 (PMC10895830; doi:10.1186/s13063-024-07967-4)
Supplement: Supplementary file 2 — Additional file 2. Description of the different categories of upper limb function classifications. Manual Ability Classification System. [file 13063_2024_7967_MOESM2_ESM.pdf]

Additional file 2: Description of the different categories of upper limb function classifications.

Manual Ability Classification System [64]

Level	Description
I	Handles objects easily and successfully
II	Handles most objects but with somewhat reduced quality and/or speed of achievement
III	Handles objects with difficulty; needs help to prepare and/or modify activities
IV	Handles a limited selection of easily managed objects in adapted situations
V	Does not handle objects and has severely limited ability to perform even simple actions

See Eliasson et al. [64] *for a more detailed description and distinction criteria between levels.*

Modified House Functional Classification System [57]

Grade	Description	Activity Level
0	Does not use	Does not use
1	Poor passive assist	Uses as stabilizing weight only
2	Fair passive assist	Can hold on to object placed in hand
3	Good passive assist	Can hold on to object and stabilize it for use by the other hand
4	Poor active assist	Can actively grasp object and hold it weakly
5	Fair active assist	Can actively grasp object and stabilize it well
6	Good active assist	Can actively grasp object and then manipulate it against other hand
7	Spontaneous use, partial	Can perform bimanual activities easily and occasionally uses the hand spontaneously
8	Spontaneous use, complete	Uses hand completely independently without reference to the other hand

See Koman et al. [57] *for a more detailed description of each grade.*
